# Evaluation of a demand-creation intervention for couples’ HIV testing services among married or cohabiting individuals in Rakai, Uganda: a cluster-randomized intervention trial

**DOI:** 10.1186/s12879-016-1720-y

**Published:** 2016-08-08

**Authors:** Joseph K. B. Matovu, Jim Todd, Rhoda K. Wanyenze, Robert Kairania, David Serwadda, Fred Wabwire-Mangen

**Affiliations:** 1 Department of Community Health and Behavioral Sciences, Makerere University College of Health Sciences, School of Public Health, Kampala, Uganda; 2 Department of Population Health, London School of Hygiene and Tropical Medicine, London, UK; 3 Department of Disease Control and Environmental Health, Makerere University College of Health Sciences, School of Public Health, Kampala, Uganda; 4 Rakai Health Sciences Program/Uganda Virus Research Institute, Kalisizo, Uganda; 5 Regional Center for Quality of Health Care, Makerere University College of Health Sciences, School of Public Health, Kampala, Uganda

**Keywords:** Demand creation, Intervention, Couples’ HCT, Uptake, Rakai, Uganda

## Abstract

**Background:**

Uptake of couples’ HIV counseling and testing (couples’ HCT) services remains largely low in most settings. We report the effect of a demand-creation intervention trial on couples’ HCT uptake among married or cohabiting individuals who had never received couples’ HCT.

**Methods:**

This was a cluster-randomized intervention trial implemented in three study regions with differing HIV prevalence levels (range: 9–43 %) in Rakai district, southwestern Uganda, between February and September 2014. We randomly assigned six clusters (1:1) to receive the intervention or serve as the comparison arm using computer-generated random numbers. In the intervention clusters, individuals attended small group, couple and male-focused interactive sessions, reinforced with testimonies from ‘expert couples’, and received invitation coupons to test together with their partners at designated health facilities. In the comparison clusters, participants attended general adult health education sessions but received no invitation coupons. The primary outcome was couples’ HCT uptake, measured 12 months post-baseline. Baseline data were collected between November 2013 and February 2014 while follow-up data were collected between March and April 2015. We conducted intention-to-treat analysis using a mixed effects Poisson regression model to assess for differences in couples’ HCT uptake between the intervention and comparison clusters. Data analysis was conducted using STATA statistical software, version 14.1.

**Results:**

Of 2135 married or cohabiting individuals interviewed at baseline, 42 % (*n* = 846) had ever received couples’ HCT. Of those who had never received couples’ HCT (*n* = 1,174), 697 were interviewed in the intervention clusters while 477 were interviewed in the comparison clusters. 73.6 % (*n* = 513) of those interviewed in the intervention and 82.6 % (*n* = 394) of those interviewed in the comparison cluster were interviewed at follow-up. Of those interviewed, 72.3 % (*n* = 371) in the intervention and 65.2 % (*n* = 257) in the comparison clusters received HCT. Couples’ HCT uptake was higher in the intervention than in the comparison clusters (20.3 % versus 13.7 %; adjusted prevalence ratio (aPR) = 1.43, 95 % CI: 1.02, 2.01, *P* = 0.04).

**Conclusion:**

Our findings show that a small group, couple and male-focused, demand-creation intervention reinforced with testimonies from ‘expert couples’, improved uptake of couples’ HCT in this rural setting.

**Trial registration:**

ClinicalTrials.gov, NCT02492061. Date of registration: June 14, 2015.

**Electronic supplementary material:**

The online version of this article (doi:10.1186/s12879-016-1720-y) contains supplementary material, which is available to authorized users.

## Background

There are renewed efforts to increase the proportion of individuals who are aware of their HIV status. In 2014, the Joint UN Program on HIV/AIDS (UNAIDS) released new targets dubbed “90-90-90”: 90 % of people living with HIV are aware of their HIV status; 90 % of people living with HIV have been enrolled into HIV care; and 90 % of people living with HIV who are enrolled in HIV care have reached viral suppression by 2020 [1]. To support the attainment of these global targets, the World Health Organization’s (WHO) recent guidance on HIV testing services [2] has identified priority populations that require urgent targeting, including couples and partners of people living with HIV. Couples’ and partner HIV testing can improve timely linkage into HIV care, support mutual disclosure, and can improve adherence to HIV treatment if one or both partners have been enrolled into HIV care [3]. Couples’ and partner HIV testing can also improve timely identification and enrolment into HIV care among men who usually report late for HIV diagnosis and consequently enroll late into HIV care [2].

Evidence from Demographic and Health Surveys [4] as well as from population-based studies [5] suggest that between one-half to two-thirds of HIV-affected married or cohabiting couples have at least one partner who is HIV-positive; but only less than 30 % of such couples are aware of their partners’ HIV status [6]. Although recent scientific evidence points to the need for immediate enrolment of HIV-positive individuals into HIV care [7, 8], individuals can only enroll into HIV care if they are tested and are aware of their own HIV status. Unfortunately, fewer couples have tested together or disclosed their HIV status to each other [6, 9, 10], presenting a missed opportunity for timely enrolment into HIV care among HIV-discordant and concordant HIV-positive couples.

Several efforts in Rwanda and Zambia [11–13] have shown that couples’ HIV testing can be increased with more targeted interventions. In both countries, efforts to invite couples to test for HIV through influential network agents have yielded positive results with a significant proportion of invited couples responding to the invitations. Recent interventions in Malawi [14, 15], Tanzania [16] and South Africa [17] which included inviting the male partner for couples’ HIV testing at prevention of mother-to-child transmission (PMTCT) of HIV or antenatal care (ANC) clinics replicate similar findings as those reported in Zambia and Rwanda, suggesting that official invitations to male partners to attend couples’ HCT with their pregnant female partners can improve uptake of couples’ HIV testing services.

However, with the exception of invitations delivered through HIV-positive women receiving Option B+ in Malawi [14] where 52–74 % of male partners presented to the clinic following the invitations and received couples’ HCT, previous ANC or PMTCT-based studies have shown that fewer than 35 % of men have honored invitations that were delivered through their female partners [17–19]. Outside ANC or PMTCT settings, efforts to increase couples’ HCT have included targeted invitations delivered to couples in the community by influential network agents as has been implemented in Rwanda and Zambia [11–13]. These efforts have resulted in improved uptake of couples’ HCT among couples “in the door way”; that is to say, among couples that have honored the invitations. However, fewer couples usually respond to the invitations: In Rwanda, only 14.3 % (*n* = 1,411) of invitations distributed in 2007 and 18 % (*n* = 4,513) of invitations distributed in 2012 were honored [11, 13]; in Zambia, only 6 % (*n* = 1,727) invitations distributed in 2012 were honored [12]. This begs the question, “*What else can be done to improve uptake of couples’ HIV testing services*?” In this study, we present the findings from an evaluation of a cluster-randomized, demand-creation intervention trial that was implemented to promote couples’ HIV counseling and testing uptake among married or cohabiting individuals in rural Rakai district, southwestern Uganda. We believe that study findings will help to shade more light on what else can be done to improve couples’ HIV testing service uptake in sub-Saharan Africa.

## Methods

### Study design

We implemented a cluster-randomized, demand creation intervention trial aimed at improving couples’ HCT uptake among married or cohabiting individuals resident in three different HIV prevalence settings in Rakai, Uganda. Study clusters are randomly selected communities within the Rakai Community Cohort Study’s (RCCS) enumeration area. The RCCS is implemented by the Rakai Health Sciences Program, a biomedical research collaboration based in Kalisizo, Rakai district. Evidence from the RCCS or the Rakai cohort in short suggest that couples’ HCT uptake can vary depending on background HIV prevalence [20]; thus, the decision to use a cluster-randomized intervention trial was based on the assumption that the intervention would perform differently in different background HIV prevalence settings.

### Study population

The study was conducted among married or cohabiting individuals (aged 15–49 years, resident in the three HIV prevalence strata) who had never received couples’ HCT, identified from the baseline study visit. At baseline, individuals were asked if they had ever tested together with any of their marital partners (including their current marital partners) and those responding in the negative were considered to have never received couples’ HCT. Individuals were considered to be “married” if they reported that they were in a religious, civil or traditionally recognized marriage; and to be “cohabiting” if they lived together as husband and wife (and the community regarded them as such) although they did not belong to any of the “officially recognized” categories of marriage mentioned above. It is important to note that while the intervention targeted couples in which both partners had never received couples’ HCT, married or cohabiting individuals were enrolled into the study in their capacity as individuals. Thus, the unit of analysis for this paper is individuals rather than couples.

### Study site

The baseline study and the subsequent intervention were implemented in three HIV prevalence strata that were selected from the eleven study regions that form the Rakai cohort. The Rakai cohort is a population-based cohort that was established in 1994 for a randomized community intervention trial of sexually transmitted diseases (STD) control for HIV prevention [21] in Rakai district, and has undergone continuous annual sero-behavioral surveillance since then. In the context of this study, we defined a study region as an area with artificially demarcated boundaries, consisting of a set of study communities/clusters brought together for purposes of research; the demarcated study regions are separated by a “buffer zone” to avoid contamination [21]. Each year, approximately 15,000 consenting individuals aged 15–49 years, resident in the ten study regions, are administered socio-demographic, behavioral and health questionnaires. Blood samples are collected for HIV serology and individuals can elect to receive their HIV test results alone or together with their partners. Previous studies in this cohort suggest that over 80 % of the residents have ever received their HIV test results [6, 22] but less than 30 % of the tested individuals have ever received their HIV test results as a couple [6].

### Randomization procedures

To facilitate the process of selecting the HIV prevalence strata, the eleven study regions were grouped into three HIV prevalence strata; i.e. *low* (9.7–11.2 %), *middle* (11.4–16.4 %) and *high* (20.5–43 %) HIV prevalence strata based on HIV prevalence data [23] from the Rakai Community Cohort Study (RCCS). Each stratum had at least three study regions; one was purposively selected to represent each stratum (i.e. Buyamba [background HIV prevalence: 9.7 %] to represent the *low HIV prevalence* stratum; Katana [background HIV prevalence: 12 %] to represent the *medium-term HIV prevalence*; and Kasensero [background HIV prevalence: 43 %] to represent the *high HIV prevalence* stratum). The selection of the study region from each stratum took into consideration the existence of other health promotional interventions within the cohort; study regions in which there were other ongoing health interventions were not selected to participate in the study. Each study region had between 3 and 8 study clusters; four of these were randomly selected to participate in the study, two as intervention and two as comparison clusters. Of the 12 clusters overall, six were randomly assigned to the intervention and six to the comparison clusters based on a ratio of 1:1 using computer-generated random numbers. The random numbers were generated by a Data Manager who was working with the Rakai Health Sciences Program at the time of the study but who was not primarily involved in the design or implementation of the study.

### Sample size determination

To estimate the sample size for the intervention, we assumed a 35 % uptake of couples’ HCT in the intervention communities compared with a baseline of 25 % in the standard of care/comparison communities [6]. We set two-sided alpha level at 0.05 and assumed a power of 90 % to detect differences in the proportion of individuals accepting couples’ HCT between the intervention and comparison communities. We used 12 study communities (i.e., 4 study communities per study region x 3 study regions) and accounted for cluster design effect using an intra-class correlation of 0.0039 [24]. Based on these assumptions, we estimated that we would need to enroll 1538 individuals in each arm (i.e. intervention and comparison communities) or 3,076 individuals overall, after adjusting for non-response rate (out-migration, refusal to participate, loss to follow-up) estimated at 15 % [25]. Sample size estimation was done using the *sampsi* [sampsi .25 .35, power (.9)] and *sampclus* [sampclus, numclus (12) rho (0.0039)] commands in STATA (STATA statistical software, version 11.0).

### Intervention in context

The design of the intervention was informed by theoretical constructs (e.g. perceived benefits, perceived barriers, readiness to receive couples’ HCT, relative advantage of couples’ versus individual HCT, among others) drawn from three commonly used behavior change theories, namely; the Health Belief Model [26], Stages of Change Model [27] and Diffusion of Innovations Theory [28]. The intervention benefitted from a baseline study on the correlates of previous couples’ HCT uptake among married individuals resident in three HIV prevalence strata [20] as well as from an earlier qualitative study conducted to explore the motivations for and barriers to couples’ HCT uptake among married individuals in Rakai district [29]. Findings from the baseline study showed that while 95 % (*n* = 2,020) of married or cohabiting individuals had ever tested for HIV, only 42 % (*n* = 846) had ever tested as a couple. We found that awareness about the availability of couples’ HCT services in the community was a significant predictor of previous couples’ HCT uptake, suggesting that any interventions aimed at promoting couples’ HCT had to improve individuals’ awareness of the availability of couples’ HCT services in the community [20].

On the other hand, findings from the qualitative study suggested that interventions aimed at improving couples’ HCT should target married or cohabiting individuals with messages on the benefits of couples’ HCT while minimizing the barriers to couples’ HCT. Participants also stressed the need to use “expert couples” that have ever tested together to give testimonies on how they managed to navigate the couples’ HCT process as well as the need to sensitize men -in their capacity as decision-makers in the relationship - about the benefits of couples’ HCT [29]. Thus, the intervention was designed to create demand for couples’ HCT uptake through a variety of approaches (hereafter defined as “the intervention”) to raise awareness about the availability and benefits of couples’ HCT services in the intervention clusters.

### Intervention description

The demand-creation intervention was designed and implemented as a small group, interactive, two-in-one strategy, comprising couple- and male-focused sessions; reinforced with testimonies from already tested couples. The intervention was implemented in a phased manner following the order in which the baseline study was conducted. That is, study regions which received the baseline visits earlier were also targeted much earlier with the intervention than those that received the baseline visits later.

The implementation of the intervention started with couple-focused sessions; and after three months, male-focused sessions were conducted. Participants had up to three months to seek couples’ HCT after the couple-focused sessions and another three months after the male-focused sessions for a total of six intervention months. This process was conducted in each of the six intervention clusters until all of them were covered (with significant overlaps between clusters). We used the term “couple-focused sessions” to refer to sessions that targeted both members of the relationship (although, in practice, some individuals attended alone rather than together with their partners) to distinguish them from “male-focused sessions” that targeted male partners alone. The two types of sessions were implemented as two components of the same strategy (the content and mode of delivery for both types of sessions was similar); individuals who attended “couple-focused sessions” could also attend “male-focused sessions” if they were men. The need to invite men as a special group was based on prior evidence that shows that men usually don’t respond to invitations to test together with their partners [29] yet in their capacity as decision-makers, their opinions could affect uptake of couples’ HCT by their female partners. In total, 52 meetings were conducted including both couple- and male-focused meetings, with an average attendance of 30 individuals - in keeping with the small group communication approach [30] adopted for the intervention.

To participate in the interactive sessions, married or cohabiting individuals received a letter of invitation from the Rakai Health Sciences Program asking them to meet at a designated venue on a particular date and time to discuss “health issues pertaining to married or cohabiting individuals”. The letters of invitation were delivered by a Community Health Mobilizer (CHM). We did not record any cases where CHMs reported that individuals refused to accept the invitations, meaning that all married or cohabiting individuals that were contacted received their letters of invitation. In situations where the individuals listed on the letter of invitation were found to have died or separated or out-migrated from the study cluster, the CHM returned the invitation to the intervention coordination office at Kalisizo (Rakai district). This was done to ensure full control over the individuals invited to the couple and male-focused sessions but most importantly, the letters were addressed to particular individuals named; so, it would not be appropriate to use them to invite other individuals.

Both couple and male-focused sessions were conducted in the form of small-group, interactive sessions lasting about 4–6 h, during which discussions were held on the fears and benefits of couples’ HIV testing, and participants were encouraged to test for HIV together with their partners. The sessions were facilitated by a senior HIV counselor with support from the lead author and one of the counseling supervisors. Participants were taken through a list of 10 items that centered on the role of couples’ HCT, how to initiate discussion about couples’ HCT with a partner, potential barriers and fears about couples’ HCT, and how these barriers can be overcome (Table 1). The topics were generated from an earlier qualitative study [29] with additional content adopted from the Uganda National Couples’ HIV Counseling and Testing Strategy [31]. The discussions were interactive and allowed participants to pose questions seeking for clarity at any point during the discussion. Previously tested couples were invited to attend these sessions (as ‘expert couples’) to help allay some of the fears and anxieties raised by the attendants, but also to give testimonies on how they negotiated the HIV testing process before they tested together as a couple. At least one “expert couple” - selected from already tested couples within each locality - was invited to attend each session although in some cases, both members of the couple could not afford to attend at once. ‘Expert couples’ in which at least one of the partners was infected with HIV shared their experiences on how they were able to cope and live together since they tested for HIV.

**Table 1:** Topics discussed during the couple- and male-focused meetings

• Current trends in HIV and AIDS in Uganda (general and couple-specific perspectives) • Current status of HIV counselling and testing uptake: national & district-level picture • HIV counselling and testing (HCT) styles: individual HCT, couples’ HCT • Introduction to couples’ HIV counselling and testing: meaning, processes and benefits • How to bring up the subject of couples’ HCT to a partner • Possible HIV test results for a couple (concordant HIV-positive, concordant HIV-negative, HIV discordance): meanings, implications and coping mechanisms • Prevention with HIV-negative couples • Prevention with HIV-positive couples • Family planning and prevention of mother-to-child transmission of HIV • Experiences from ‘expert couples’	

**Fig. 1:**
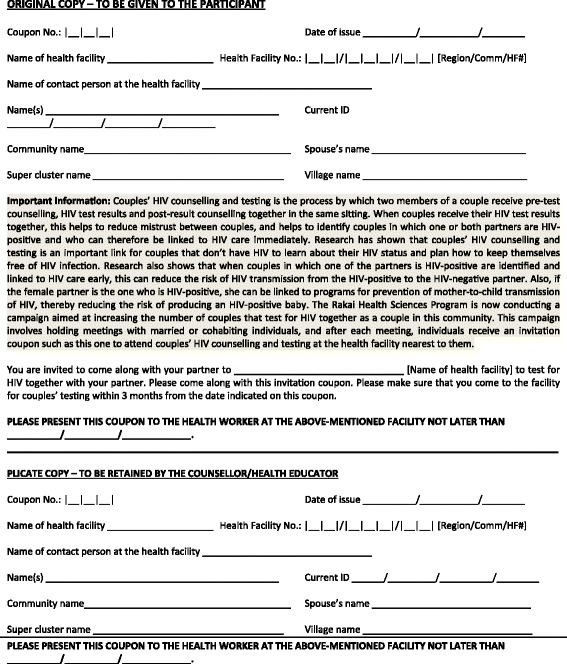
Couple invitation coupon

At the end of each [couple- or male-focused] session, individuals received a ‘*couple invitation coupon*’ (Fig. 1) that invited them to test for HIV together at a designated health facility in the community. Individuals who attended the sessions alone - either because their marital partner declined to attend or because they were men (who were invited alone to male-focused sessions) - were given two coupons; one for themselves and the other for their partner. Each coupon consisted of two parts; the upper part that the HIV counselor retained and the lower part that was given to the individuals to take to the HIV testing facility. Each coupon had an identification number for easy tracking and contained messages on the benefits of testing as a couple; including the possibility of being enrolled into HIV care immediately if one or both partners tested HIV-positive. Although the primary purpose of the intervention was to promote couples’ HCT, individuals were free to test alone or together with their partners. Those that opted to test alone were encouraged to disclose their HIV status to their marital partners, in line with the Uganda HCT Policy [32]. On a monthly basis (for up to three months), a senior counselor from the Rakai Health Sciences Program went to the designated health facility to pick all coupons for those who had tested within the month. These coupons were entered into a tracking database at the main research station in Kalisizo, Rakai district and later used to determine the number of individuals responding to the intervention. The intervention was implemented between February and September 2014.

### Promotion of HIV counseling and testing services in the comparison clusters

Six communities served as the comparison clusters. Within these communities, HIV counseling and testing was offered as one of the general HIV services provided to the community by the Rakai Health Sciences Program. No specific invitations were sent out to invite married or cohabiting individuals to test together as a couple at any of the participating health facilities. There were no special sessions for men-only or couples either. Instead, general health education services were provided through town-hall meetings or local theatre targeting entire communities as the standard of care. The general health education sessions were conducted using a pre-existing “mobilization guide” or “*Mob Guide*” that was used by the Rakai Health Sciences Program (RHSP) for community mobilization and health education purposes at the time. The sessions were conducted at about the same time when the intervention was ongoing, and were usually held in the evenings between 4 and 6 pm; the time that community residents found convenient to attend such sessions. The sessions in the comparison clusters targeted all community residents within a given cluster rather married or cohabiting individuals per se, and were attended by people of all age-groups, including children, young people and adults. The content of the messages was based on RHSP’s risk reduction and HIV prevention messages including testing for HIV and other sexually transmitted diseases. While HIV messages provided to the participants included the need for HIV testing, including couples’ HIV testing, it was up to the attending individuals to go to the designated health facility to test for HIV either alone or together with their partners.

### Data collection procedures and methods

Baseline data were collected between November 2013 and February 2014 while follow-up data were collected between March and April 2015 (twelve months post-baseline). This was done to allow for married or cohabiting individuals who were reached by the intervention towards the end of the intervention period to make a decision about attending couples’ HCT at designated HIV testing facilities in the community. At the baseline and follow-up visits, data were collected using same-sex interviewers who administered paper-based questionnaires to study respondents at designated “study hubs” or at the respondents’ homes. The use of same-sex interviewers was aimed at minimizing bias in reporting of sexually sensitive information including outside sexual partners. Prior to the interviews, individuals were invited to the study hub where interviews took place between 9.00 am and 5.00 pm on the day of interview. The study team conducted interviews at each hub for 3–4 days or until when all the invited respondents had been interviewed. Individuals who were invited but did not come to the hub were followed up in their homes with the assistance of a community health mobilizer. Individuals who were not found at home were revisited at two different occasions, and if they were not found at home at these visits, they were declared as not being available for interview. Individuals who refused to be interviewed were not followed up for any subsequent visits.

Data were collected on socio-demographic (age, sex, marital status, marital order (i.e. whether or not the respondent was in the first marriage ever; or whether they had a marriage relationship that ended and were in the second, third or higher order marriage), religious affiliation) and behavioral characteristics (prior HIV testing, uptake of individual or couples’ HCT post-intervention, number of sexual partners in the past year, condom use in the past year, and current non-marital sexual relationships). We used baseline data to generate a new variable, “prior mutual HIV status disclosure at baseline” within the follow-up dataset to determine what proportion of individuals followed up reported that they disclosed their HIV status to their marital partners at baseline. This was based on our earlier observation that HIV status disclosure was closely associated with prior couples’ HCT uptake in Rakai [20]. Individuals were classified as having had prior mutual HIV status disclosure at baseline if both partners reported that they had ever disclosed their HIV status to each other at the baseline interview. Each interview took about 1 h, on average, to complete. All completed questionnaires were edited in the field to ensure completeness and accuracy of data collected. Edited questionnaires were transported to the Rakai Health Sciences Program field offices at Kalisizo where data entry took place. The interviews were conducted by trained Social Science graduates with long-term experience in quantitative data collection.

### Study outcomes

The primary outcome was uptake of couples’ HCT among married or cohabiting individuals with no prior couples’ HCT experience who were resident in the intervention or comparison clusters. Couples’ HCT uptake was defined as self-reported receipt of HCT by two married or cohabiting individuals in a heterosexual relationship at the same sitting, expressed as a proportion of all individuals that were interviewed at the follow-up visit within each study arm. The timeline for the primary outcome was 12 months post-baseline.

### Statistical analysis

We computed descriptive analyses to determine the characteristics of respondents enrolled in the intervention and comparison clusters and conducted Chi Square tests to assess for any statistical differences between the two groups. We then compared the characteristics of married or cohabiting individuals who received couples’ HCT in each arm (out of all those who received HCT during the intervention period) by socio-demographic and behavioral characteristics using Chi Square tests. After the descriptive analyses, we conducted bivariate analysis to assess for any independent association between the primary outcome and exposure to the intervention. This analysis was extended to include explanatory variables including all the socio-demographic and behavioral characteristics. Only one explanatory variable (prior mutual HIV status disclosure at baseline) was found to be significant (*P* < 0.05) at the bivariate analysis. This was automatically considered for the multivariable analysis. In addition, we included suspected confounders (sex, marital duration, number of extra-marital partners reported in the past 12 months; condom use in the past 12 months, self-reported current extra-marital relationships, and HIV status at baseline) even if they were not significantly associated with couples’ HCT at the bivariate analysis. This was based on prior evidence from other studies that showed a close association between these covariates and couples’ HCT; e.g. marital duration [20] or on biological plausibility considerations. For instance, individuals reporting extra-marital partners would ideally be less likely to test as a couple for fear that such testing could reveal cases of hidden infidelity [33]. We prepared the dataset for panel-level analysis using the *xtset* command in STATA and conducted intention-to-treat analysis using a mixed effects Poisson regression model to assess for differences in couples’ HCT uptake between the intervention and comparison clusters, after adjusting for potential and suspected confounders. We estimate that this study had a post-hoc statistical power of 73.8 % to detect a prevalence ratio of 1.43 as significant at an alpha-level of 0.05 when comparing couples’ HCT uptake in the intervention to couples’ HCT uptake in the comparison clusters. Data analysis was conducted using STATA statistical software, version 14.1. We report the findings in accordance with the CONSORT 2010 statement (Additional file 1) [34]. This trial is registered with ClinicalTrials.gov, NCT02492061.

### Ethical considerations

The protocol for the demand-creation intervention trial was cleared by the Higher Degrees, Research and Ethics Committee of Makerere University School of Public Health (IRB00011353) and approved by the Uganda National Council for Science and Technology. All participants gave written informed consent prior to participating in the study.

## Results

### Baseline characteristics

**Fig. 2:**
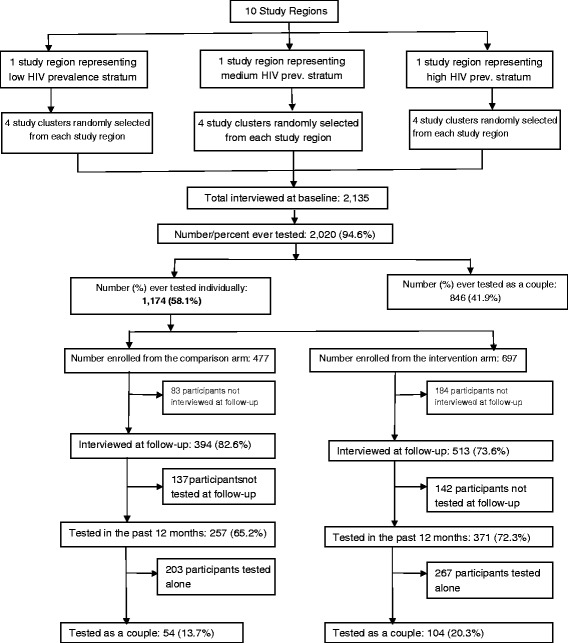
Trial profile

Figure 2 shows the trial profile. At baseline, 2135 individuals were interviewed, representing 69.4 % of the targeted sample. Of those that were not interviewed (*n* = 941), 72 % did not show up at the interview location and were not traceable at home despite multiple attempts to locate them; 14 % refused to participate; 12 % had out-migrated from the community, while 2 % were found to have died prior to the interview. Of those interviewed (*n* = 2135), 2020 (94.6 %) had ever tested for HIV; 846 (41.9 %) had ever tested as a couple while 58.1 % (*n* = 1174) had never tested as a couple. Of those that had never tested as a couple, 697 were interviewed in the intervention clusters while 477 were interviewed in the comparison clusters. These individuals were the focus of the demand-creation intervention that is described in this paper.

Table 2 shows the baseline characteristics of the 1174 individuals stratified by the exposure of interest. At baseline, a slightly higher proportion of married or cohabiting individuals in the comparison than in the intervention clusters (88.2 % vs. 79.2 %, *P* < 0.0001) had lived together for 5 or more years. Individuals enrolled in the intervention clusters were better educated than those in the comparison clusters (post-primary education: 28.7 % vs. 21.6 %, *P* = 0.02). In addition, significantly more individuals in the intervention clusters reported current non-marital sexual relationships than those in the comparison clusters (15.5 % vs. 10.7 %, *P* = 0.02) but there was no significant difference in ever-use of condoms among those enrolled in the intervention (64.3 %) when compared to those enrolled in the comparison clusters (59.1 %; *P* = 0.07).

**Table 2:** Baseline characteristics of married or cohabiting individuals with no prior couples’ HCT experience, stratified by the exposure of interest

Characteristic	Comparison (*n* = 477)	Intervention (*n* = 697)	Total (*n* = 1174)	*P*-value
Study region				<0.0001
Buyamba	129 (48.9)	266 (38.2)	395 (33.6)	
Katana	233 (27.0)	179 (25.7)	412 (35.1)	
Kasensero	115 (24.1)	252 (36.1)	367 (31.3)	
Sex				0.59
Female	249 (52.2)	375 (53.8)	624 (53.1)	
Male	228 (47.8)	322 (46.2)	550 (46.9)	
Age-group				0.22
15–24	64 (13.4)	118 (16.9)	182 (15.5)	
25–34	220 (46.1)	319 (45.8)	539 (45.9)	
35+	193 (40.5)	260 (37.3)	453 (38.6)	
Marital duration				<0.0001
1–2 years	22 (4.6)	56 (8.0)	78 (6.6)	
3–4 years	35 (7.3)	89 (12.8)	124 (10.6)	
5+ years	420 (88.1)	552 (79.2)	972 (82.8)	
Marital order				0.01
1^st^	343 (71.9)	442 (63.4)	785 (66.9)	
2^nd^	105 (22.0)	200 (28.7)	305 (26.0)	
3^rd^ or higher	29 (6.1)	55 (7.9)	84 (7.1)	
Education				0.02
None	26 (5.4)	41 (5.9)	67 (5.7)	
Primary	348 (73.0)	456 (65.4)	804 (68.5)	
Post-primary	103 (21.6)	200 (28.7)	303 (25.8)	
Number of sex partners (past 12 months)				0.25
1	382 (80.1)	533 (76.5)	915 (77.9)	
2+	95 (19.9)	164 (23.5)	259 (22.1)	
Condom use (ever)				0.07
No	195 (40.9)	249 (35.7)	444 (37.8)	
Yes	282 (59.1)	448 (64.3)	730 (62.2)	
Current non-marital sex				0.02
No	426 (89.3)	589 (84.5)	1015 (86.5)	
Yes	51 (10.7)	108 (15.5)	159 (13.5)	
HIV status at baseline				<0.0001
HIV negative	405 (84.9)	527 (75.6)	932 (79.4)	
HIV positive	10 (2.1)	86 (12.3)	96 (8.2)	
HIV results not available	62 (13.0)	84 (12.1)	146 (12.4)	
HIV status disclosure (ever)				0.65
Neither disclosed	227 (47.6)	336 (48.2)	563 (48.0)	
Only one disclosed	197 (41.3)	295 (42.3)	492 (41.9)	
Both ever disclosed to each other	53 (11.1)	66 (9.5)	119 (10.1)	
Time since last tested for HIV				0.02
Less than 1 year	276 (57.9)	455 (65.3)	731 (62.3)	
1 year	60 (12.6)	82 (11.7)	142 (12.1)	
2+ years	141 (29.5)	160 (23.0)	301 (25.6)	

Overall, 8.2 % of those enrolled in the study were HIV-positive; 79.4 % were HIV-negative while HIV status information was not available for 12.4 % of the respondents. Majority of the individuals (*n* = 731, 62.3 %) reported that they last tested for HIV less than a year prior to interview; 142 (12.1 %) reported that they last tested one (1) year prior to interview; while 301 (25.6 %) last tested for HIV two or more years prior to interview. Individuals in the comparison clusters were less likely to have tested in less than a year (57.9 % vs. 65.3 %, *P* = 0.02); however, a higher proportion of individuals in the comparison clusters (12.7 %) reported that they last tested for HIV in one year’s time than those in the intervention clusters (11.7 %). At baseline, 48 % reported that they had never disclosed their HIV status to any of their sexual partners, 41.9 % reported that only one of the partners had ever disclosed their HIV status to their other partner while 10.1 % reported that they had ever disclosed their HIV status to each other (i.e. had prior mutual HIV status disclosure). However, there was no significant difference in terms of prior HIV status disclosure between those enrolled in the comparison versus those enrolled in the intervention clusters (*P* = 0.65). There were no significant differences between individuals enrolled in the comparison and those enrolled in the intervention clusters in terms of gender (*P* = 0.59), age-group (*P* = 0.22), number of sexual partners in the past year (*P* = 0.25) and ever-use of condoms for HIV prevention (*P* = 0.07).

### Follow-up characteristics

Nine hundred seven individuals (77.2 %) were interviewed at follow-up; 73.6 % (*n* = 513) in the intervention clusters and 82.6 % (*n* = 394) in the comparison clusters (Fig. 2). Overall, 267 (22.7 %) of individuals interviewed at baseline (i.e., 17.4 % in the comparison clusters and 26.4 % in the intervention clusters) were not interviewed at follow-up largely due to failure to trace them at the time of interview (80 % of those not interviewed did not turn up at the study hub or were not at home at the time of interview; the remaining 20 % were not interviewed either because they had migrated outside the study cluster, were dead; separated or divorced; or they refused to participate in the follow-up interview). Of those interviewed at follow-up, 628 (69.2 %) reported that they tested for HIV and received their HIV test results during the intervention period; 257 (65.2 %) in the comparison clusters and 371 (72.3 %) in the intervention clusters. Of the 628 who received HCT during the intervention period, 311 (49.5 %) received their HIV results from the participating health facilities; 188 (29.9 %) received their results from the Rakai Health Sciences Program, 68 (10.8 %) from Uganda Cares (a Non-Government Organization that offers HCT and antiretroviral therapy in the study district) while 61 (9.7 %) received their HIV results from private health facilities.

Table 3 shows differences in couples’ HCT uptake by background characteristics, stratified by the exposure of interest. Overall, couples’ HCT uptake was significantly higher in the intervention arm than in the comparison arm (20.3 % versus 13.7 %, *P* = 0.01). Compared to individuals in the comparison arm, those in the intervention arm were significantly more likely to receive couples’ HCT if they were: males (23.1 % versus 14.4 %, *P* = 0.02), aged 30-39 years (22.3 % versus 11.4 %, *P* = 0.003); had post-primary education (21.7 % versus 10.5 %, *P* = 0.04) and had stayed in their marital unions for 3–4 years (33.3 % versus 10.3 %, *P* = 0.02). In addition, compared to those in the comparison arm, individuals in the intervention were significantly more likely to receive couples’ HCT if they reported prior mutual HIV status disclosure at baseline (28.8 % versus 18.8 %, *P* = 0.03); reported only one sexual partner in the past 12 months (21.5 % versus 14.9 %, *P* = 0.03), reported no condom use in the past 12 months (19.1 % versus 12.7 %, *P* = 0.02), reported no current non-marital sexual relationship (21.5 % versus 14.3 %, *P* = 0.01) and were in their first-ever (first-order) marriage (21 % versus 13 %, *P* = 0.01).

**Table 3:** Couples’ HCT uptake among married or cohabiting individuals by the exposure of interest and background characteristics

Characteristic	Comparison arm		Intervention arm		*P*-value*
	*N* = 394	n (%)	*N* = 513	n (%)	
Study region					
Buyamba	113	16 (14.2)	217	38 (17.5)	0.43
Katana	188	20 (10.6)	144	17 (11.8)	0.74
Kasensero	93	18 (19.3)	152	49 (32.2)	**0.03**
Sex					
Female	206	27 (13.1)	266	47 (17.7)	0.18
Male	188	27 (14.4)	247	57 (23.1)	**0.02**
Age-group					
19–29	105	18 (17.1)	138	34 (24.6)	0.16
30–39	193	22 (11.4)	233	52 (22.3)	**0.003**
40+	96	14 (14.6)	142	18 (12.7)	0.67
Education					
None	24	03 (12.5)	30	09 (30.0)	0.12
Primary	294	43 (14.6)	345	65 (18.8)	0.16
Post-primary	76	08 (10.5)	138	30 (21.7)	**0.04**
Marital duration					
≤2 years	18	04 (22.2)	37	09 (24.3)	0.86
3–4 years	29	03 (10.3)	63	21 (33.3)	**0.02**
5+ years	347	47 (13.5)	413	74 (17.9)	0.10
Prior mutual HIV status disclosure at baseline					
Neither partner disclosed	164	14 (8.5)	180	27 (15.0)	0.06
Only one partner disclosed	81	12 (14.8)	142	22 (15.5)	0.89
Both partners disclosed	149	28 (18.8)	191	55 (28.8)	**0.03**
No. of sexual partners in past 12 months					
1	309	46 (14.9)	396	85 (21.5)	**0.03**
2+	85	08 (9.4)	117	19 (16.2)	0.16
Condom use in past 12 months					
No	314	40 (12.7)	383	73 (19.1)	**0.02**
Yes	80	14 (17.5)	130	31 (23.8)	0.28
Current non-marital sex					
No	321	46 (14.3)	428	92 (21.5)	**0.01**
Yes	73	08 (11.0)	85	12 (14.1)	0.55
Marital order					
First	276	36 (13.0)	333	70 (21.0)	**0.01**
Second	95	15 (15.8)	151	29 (19.2)	0.50
Third or higher	23	03 (13.0)	29	05 (17.2)	0.68

*P-value based on two-sample test of proportions; *p*-values that are less than 0.05 are shown in bold type

Overall, couples’ HCT uptake significantly differed by background HIV prevalence setting: couples’ HCT was 11.4 % (*n* = 37) in the medium HIV prevalence stratum; 16.4 % (*n* = 54) in the low HIV prevalence stratum and 27.3 % (*n* = 67) in the high HIV prevalence stratum (*P* < 0.0001). However, when couples’ HCT uptake was stratified by exposure of interest (i.e. intervention versus comparison clusters), we found no significant difference in couples’ HCT uptake between those in the intervention and comparison clusters in the low (17.5 % versus 14.3 %, *P* = 0.43) and medium (11.8 % versus 10.6 %, *P* = 0.74) HIV prevalence strata (Table 3).

### Effect of the demand-creation intervention on couples’ HCT uptake

Table 4 shows the unadjusted and adjusted prevalence ratios (PR) and 95 % confidence intervals (CI) associated with couples’ HCT uptake among individuals with no prior couples’ HCT. As shown, uptake of couples’ HCT was significantly higher in the intervention clusters than in the comparison clusters (Adjusted Prevalence Ratio [Adj. PR] =1.43, 95 % CI, 1.02, 2.01; *P* = 0.04). Couples’ HCT uptake was associated with being male (Adj. PR = 1.41, 95 % CI, 1.00, 1.98; *P* = 0.05), condom use in the past year (Adj. PR = 1.87, 95 % CI, 1.25, 2.79, *P* = 0.002) and prior mutual disclosure of HIV status at baseline (Adj. PR = 1.99, 95 % CI, 1.36, 2.91, *P* < 0.0001). Reporting 2 or more extra-marital sexual partners in the past year was significantly associated with less likelihood of receiving couples’ HCT (Adj. PR = 0.46, 95 % CI, 0.27, 0.78, *P* = 0.004).

**Table 4:** Unadjusted and adjusted prevalence ratios and 95 % confidence intervals associated with couples’ HCT uptake among married or cohabiting individuals with no prior couples’ HCT in Rakai, Uganda

Variable	Unadjusted Prevalence Ratio (PR) and 95 % Confidence Intervals (CI)	Adjusted PR and 95 % CI^a^
Study arm		
Comparison	1.00	1.00
Intervention	1.33 (0.83, 2.12)	1.43 (1.02, 2.01)
Sex		
Female	1.00	1.00
Male	1.22 (o.89, 1.67)	1.41 (1.00, 1.98)
Marital duration		
<=2 years	1.00	1.00
3–4 years	1.10 (0.56, 2.17)	1.19 (0.60, 2.34)
5+ years	0.80 (0.44, 1.45)	0.79 (0.44, 1.42)
No. of sexual partners in past year		
1	1.00	1.00
2+	0.71 (0.47, 1.07)	0.46 (0.27, 0.78)
Condom use in the past year		
No	1.00	1.00
Yes	1.25 (0.88, 1.78)	1.87 (1.25, 2.79)
Current non-marital sex		
No	1.00	1.00
Yes	0.69 (0.43, 1.10)	0.68 (0.40, 1.17)
Prior Mutual HIV disclosure (at baseline)		
Neither disclosed	1.00	1.00
Only disclosed	1.23 (0.78, 1.94)	1.23 (0.78, 1.94)
Both disclosed	2.01 (1.29, 3.12)	1.99 (1.36, 2.91)
HIV status at baseline		
HIV-positive	1.00	1.00
HIV-negative	1.63 (0.82, 3.22)	1.56 (0.80, 3.04)
HIV status not available	1.46 (0.57, 3.75)	1.38 (0.56, 3.41)

^a^Adjusted for sex, marital duration, prior HIV status disclosure (at baseline), number of sexual partners in the past 12 months; condom use in the past 12 months, current non-marital sexual relationships and HIV status at baseline

## Discussion

Our study which evaluated the effect of the demand-creation intervention trial that was implemented to improve uptake of couples’ HCT among married or cohabiting individuals with no prior couples’ HCT experience showed that, compared to the standard of care, the intervention increased couples’ HCT by 43 % after adjusting for potential and suspected confounders. These findings suggest that the use of small group, couple and male-focused interactive sessions reinforced with testimonies from ‘expert couples’, can improve couples’ HCT uptake in this rural setting. Similar results have been reported from other couple-focused interventions implemented in other countries [11–13, 17] suggesting that innovative couple-focused interventions targeting married or cohabiting individuals can enhance uptake of couples’ HCT particularly in those who have never tested as a couple. However, it is important to note that uptake of couples’ HCT in the intervention arm was rather modest (20.3 %) despite the use of multiple strategies to improve couples’ HCT uptake in this population. The modest couples’ HCT uptake could have been due to the fact that some individuals who attended the intervention might have failed to travel to the designated health facilities due to lack of money for transport or could simply have gained limited motivation to test together with their marital partners despite attending the intervention. Either way, our findings suggest a need for alternative interventions that can improve couples’ HCT uptake beyond the levels registered in our intervention. These alternative interventions might include provision of on-site, rapid couples’ HCT where individuals attending the interventions are tested together on site; or provision of HIV self-test kits for individuals to test themselves at home [35, 36]. As Kumwenda et al. [35] have noted, HIV self-testing can offer a convenient and confidential way for both partners to test together for HIV since it can be conducted outside formal health facilities, including at home [37].

The finding that couples’ HCT uptake was associated with being male is a striking one given that male participation in HIV prevention, care and treatment programs has always been suboptimal [18, 38] and suggest that our efforts to enhance male participation through male-focused sessions yielded positive results. The design of the intervention to include male-focused sessions was informed by earlier findings from an earlier qualitative study [29] in which participants recommended the need to convene special sessions targeting men (in their capacity as decision-makers in the home) in order to improve male participation in HCT. Evidence from male-focused interventions, conducted elsewhere, suggests that targeting male partners can enhance uptake of couples’ HCT and other HIV prevention services [14–17, 39]. For instance, in Malawi, Rosenberg et al. [14] found that enhanced invitations (invitations plus tracing of male partners who did not present at the clinic) targeting male partners of HIV-positive pregnant women who were enrolled into an Option B+ program, resulted in increased uptake of couples’ HCT when compared to invitations alone. In an earlier study, Nyondo et al. [15] found that the use invitation cards enhanced male partner involvement in Prevention of Mother-to-Child Transmission of HIV program in Blantyre, Malawi. Similar results have been reported in other community-based interventions in Rwanda [11, 13], Zambia [12] and South Africa [17, 40], among other countries, suggesting that innovative approaches that aim to target men or entire couples can improve uptake of couples’ HCT services. The findings from our study, conducted among married or cohabiting individuals in a rural population-based cohort, add further credence to these earlier studies by confirming that male participation in HCT programs can be enhanced through male-focused interventions implemented outside formal healthcare settings.

We have also reported that couples’ HCT was strongly associated with prior mutual disclosure of HIV status at baseline. HIV disclosure between partners can support mutual discussion of HIV/AIDS and its implications on the family setting, thereby providing an opportunity to enhance communication about HIV/AIDS, including the need for HCT. Evidence suggests that communication between partners about HIV/AIDS in general and couples’ HCT in particular can enhance uptake of couples’ HCT among married or cohabiting individuals [41, 42]. A study by Rosenburg et al. [14] found that women who already knew their male partners’ HIV status were more likely to present with them for couples’ HCT than those who did not know about their male partners’ HIV status. Thus, it is likely that individuals who had previously disclosed their HIV results to each other (at baseline) were more motivated to test together as a couple during the intervention than those who had not done so. These findings highlight the potential role of previous mutual HIV status disclosure in improving couple communication about HIV/AIDS, thereby increasing the prospects for married or cohabiting individuals to test together for HIV.

Finally, the observation that couples’ HCT was significantly lower in individuals reporting 2 or more partners could be a result of fear of revealing hidden infidelity especially if those individuals reporting multiple sexual partners were in monogamous marital relationships (i.e. with only one marital partner). Since up to 60 % of those who reported 2 or more sexual partners were in a monogamous marital relationship (i.e. reported one marital partner; data not shown), this suggests that majority of those reporting 2 or more partners were indeed engaged in extra-marital relationships. Individuals reporting multiple sexual partners (including extra-marital relationships) may be less likely to receive couples’ HCT for fear that testing together could prove their hidden infidelity [29, 43].

Our study had several weaknesses and strengths. The findings reported in this study were obtained from self-reports of individuals who were interviewed at follow-up rather from actual coupons retrieved from the participating health facilities as we had previously planned. We were unable to use coupons retrieved from these health facilities due to the fact that some testers opted to test for HIV at other facilities elsewhere. It is likely that these self-reports might not be accurate given the problems associated with recall bias. However, we tried to minimize recall bias by asking questions that made specific reference to the intervention (in the intervention clusters) or the general health education (in the comparison clusters), thereby increasing the possibility for respondents to recall if they actually tested for HIV following exposure (or non-exposure) to the intervention.

The study was implemented in an area where previous couples’ HCT was already higher (42 %) than reported in many other settings [20]. The higher uptake of previous couples’ HCT was likely due to ongoing health education activities conducted in the study communities by the Rakai Health Sciences Program. The Rakai Health Sciences Program has been conducting annual HIV and reproductive health surveys in Rakai district since 1994 and prior to each study visit, communities receive health messages and mobilization messages that include messages on HIV testing. It is likely that individuals enrolled in the comparison clusters already had exposure to the benefits of couples’ HCT through the ongoing health education activities and went for couples’ testing during the intervention period in much the same way as individuals in the intervention clusters. Thus, our findings should be interpreted with caution since they may not be completely generalizable to other married or cohabiting individuals elsewhere.

Our intervention, which was implemented through a two-component strategy comprising couple- and male-focused sessions, appear to be rather intensive and costly given that we conducted multiple demand-creation activities (i.e. couple-focused meetings; male-focused meetings; use of “expert couples” and distribution of couple invitation coupons) across the intervention clusters. This might affect the adoption and eventual scale-up of this intervention especially in resource-constrained settings such as sub-Saharan Africa yet these settings are home to many individuals that are not aware of each other’s status including married or cohabiting individuals. One way in which these costs could be minimized is to target larger groups of married or cohabiting individuals with demand-creation activities that are reinforced with opportunities for on-site, rapid HIV testing and linkage to HIV care, including use of HIV self-testing or point-of-care HIV testing services [44]. Both approaches have the potential to increase real-time uptake of HIV testing services and can improve timely linkage to HIV care [45]. Unfortunately, at the time we implemented this study, HIV self-testing was not yet recommended as a method of HIV testing [46] and there were no point-of-care HIV testing services available in the study communities. Future research should explore the potential for these innovative testing approaches to improve HIV testing among married or cohabiting individuals including the potential to increase uptake of partner or couples’ HIV testing services among individuals with no prior couples’ HCT uptake.

The other limitation was the high loss to follow-up, with nearly a quarter (23 %) of the respondents not interviewed at follow-up. Losses to follow-up were largely due to our inability to trace up respondents in the community coupled with a limited budget that did not allow us to make multiple call-back visits to interview those missed at the earlier visits. Loss to follow-up was significantly higher in the intervention than in the comparison arm (26.4 % versus 17.4 %, *P* = 0.02). This differential loss to follow-up is likely to affect the observed effect of the intervention especially if those lost to follow-up were significantly different from those that were actually interviewed in terms of uptake of couples’ HCT. In a sub-group analysis of those lost to follow-up versus those interviewed at follow-up (data not shown), we found that there were no significant differences in the two groups by age-group, sex, education, time since last tested and prior mutual HIV status disclosure at baseline. However, the two groups differed by marital duration and study region of residence. Our findings should thus be interpreted with this loss to follow-up in mind. Nevertheless, while the loss to follow-up that is reported in this paper is much higher than that reported in a previous study in the same cohort [25]; the reported loss to follow-up is within the range (25–40 %) reported from other population-based studies in Rakai [21, 23, 47] and elsewhere [48, 49], suggesting that our study is comparable to other population-based studies.

Finally, this being a cluster-randomized intervention trial, it is evident that we did not have balanced clusters at the time of randomization thereby creating a potential for residual confounding. While we adjusted the intervention effect for both potential and suspected confounders, it is likely that there are other covariates that we did not adjust for either because we did not collect data on them or because they were not found to be significant at the bivariate analysis. The presence of residual confounding is likely to affect our ability to observe the true effect of the intervention [50]. Our findings should thus be interpreted with this limitation in mind.

Despite these limitations, our intervention combines the power of small-group, interactive sessions reinforced with testimonies from “expert couples”, thereby providing an opportunity for promoting couples’ HIV testing services in a situation where individuals interact with previously tested couples and have the opportunity of asking questions in small groups. The use of the small group approach has been found to be helpful in improving the uptake of other interventions [51, 52] and could therefore have the potential to improve uptake of couples’ HCT services if well maximized. In addition, participants in the intervention clusters received couple invitation coupons to attend HCT at designated health facilities in the study regions. Separately, each component of the intervention has been shown to enhance demand for and utilization of health services (e.g. use of invitation coupons has been shown to increase male participation in antenatal-based couples’ HCT [16, 17]); thus, the intervention maximized the combined strengths of the different components while minimizing the weaknesses associated with individual components. These approaches can be enhanced with more innovative HIV testing approaches including HIV self-testing and point-of-care HIV testing to improve the proportion of married or cohabiting individuals who can be tested together and eventually linked to appropriate HIV prevention, care and treatment services.

## Conclusion

Our findings show that a small group, couple and male-focused, demand-creation intervention, reinforced with testimonies from already tested couples improved uptake of couples’ HCT in this rural setting. Couples’ HCT uptake was associated with being male and prior mutual HIV status disclosure between partners. These findings suggest a need to incorporate small-group, couple and male-focused sessions in interventions targeted at individuals with no prior couples’ HCT uptake alongside mutual HIV status disclosure to improve uptake of couples’ HCT services. Given the intensity of the campaign and the likely high operational costs associated with the intervention, a modified version of this intervention can make use of new HIV testing technologies including HIV self-testing and point-of-care testing to improve real-time couples’ HCT uptake while minimizing operational costs.

## Abbreviations

ANC, Antenatal clinic; CHM, community health mobilizer; HCT, HIV counseling and testing; HIV, Human Immunodeficiency Syndrome; OR, odds ratio; PMTCT, Prevention of Mother-to-Child Transmission of HIV; RCCS, Rakai Community Cohort Study; STD, sexually transmitted disease

## Additional file

Additional file 1: CONSORT Statement. (DOC 218 kb)

## References

[1] UNAIDS (2014). 90-90-90: An ambitious treatment target to help end the AIDS epidemic.

[2] World Health Organization (2015). Consolidated guidelines on HIV testing services 2015.

[3] World Health Organization (2012). Guidance on couples’ HIV testing and counseling including antiretroviral therapy for treatment and prevention in sero-discordant couples: recommendations for a public health approach.

[4] de Walque D (2007). Sero-discordant couples in five African countries: implications for prevention strategies. Popul Dev Rev.

[5] Lingappa JR, Lambdin B, Bukusi EA, Ngure K, Kavuma L, Inambao M, Kanweka W, Allen S, Kiarie JN, Makhema J (2008). Regional differences in prevalence of HIV-1 discordance in Africa and enrollment of HIV-1 discordant couples into an HIV-1 prevention trial. PLoS One.

[6] Matovu JK, Denison J, Wanyenze RK, Ssekasanvu J, Makumbi F, Ovuga E, McGrath N, Serwadda D (2013). Trends in HIV counseling and testing uptake among married individuals in Rakai, Uganda. BMC Public Health.

[7] The INSIGHT START Study Group (2015). Initiation of antiretroviral therapy in early asymptomatic HIV infection. N Engl J Med.

[8] The TEMPRANO ANRS 12136 Study Group (2015). A trial of early antiretrovirals and isoniazid preventive therapy in Africa. N Engl J Med.

[9] Kenyon CR, Kirungi W, Kaharuza F, Buyze J, Bunnell R (2015). Who knows their partner’s HIV status? results from a nationally representative survey in Uganda. J Acquir Immune Defic Syndr.

[10] Doherty IA, Myers B, Zule WA, Minnis AM, Kline TL, Parry CD, El-Bassel N, Wechsberg WM. Seek, test and disclose: knowledge of HIV testing and serostatus among high-risk couples in a South African township. Sex Transm Infect. 2016;92(1):5-11 10.1136/sextrans-2014-051882 PMC13718590 26175479

[11] Allen S, Karita E, Chomba E, Roth DL, Telfair J, Zulu I, Clark L, Kancheya N, Conkling M, Stephenson R (2007). Promotion of couples’ voluntary counselling and testing for HIV through influential networks in two African capital cities. BMC Public Health.

[12] Wall KM, Kilembe W, Nizam A, Vwalika C, Kautzman M, Chomba E, Tichacek A, Sardar G, Casanova D, Henderson F (2012). Promotion of couples’ voluntary HIV counselling and testing in Lusaka, Zambia by influence network leaders and agents. BMJ Open.

[13] Wall K, Karita E, Nizam A, Bekan B, Sardar G, Casanova D, Joseph Davey D, De Clercq F, Kestelyn E, Bayingana R (2012). Influence network effectiveness in promoting couples’ HIV voluntary counseling and testing in Kigali. Rwanda. AIDS..

[14] Rosenberg NE, Mtande TK, Saidi F, Stanley C, Jere E, Paile L, Kumwenda K, Mofolo I, Ng’ambi W, Miller WC (2015). Recruiting male partners for couple HIV testing and counselling in Malawi’s option B+ programme: an unblinded randomised controlled trial. Lancet HIV.

[15] Nyondo AL, Choko AT, Chimwaza AF, Muula AS (2015). Invitation cards during pregnancy enhance male partner involvement in prevention of mother to child transmission (PMTCT) of human immunodeficiency virus (HIV) in Blantyre, Malawi: a randomized controlled open label trial. PLoS One.

[16] Jefferys LF, Nchimbi P, Mbezi P, Sewangi J, Theuring S (2015). Official invitation letters to promote male partner attendance and couple voluntary HIV counselling and testing in antenatal care: an implementation study in Mbeya Region, Tanzania. Reprod Health.

[17] Mohlala BK, Boily MC, Gregson S (2011). The forgotten half of the equation: randomized controlled trial of a male invitation to attend couple voluntary counselling and testing. AIDS.

[18] Byamugisha R, Astrom AN, Ndeezi G, Karamagi CA, Tylleskar T, Tumwine JK (2011). Male partner antenatal attendance and HIV testing in eastern Uganda: a randomized facility-based intervention trial. J Int AIDS Soc.

[19] Farquhar C, Kiarie JN, Richardson BA, Kabura MN, John FN, Nduati RW, Mbori-Ngacha DA, John-Stewart GC (2004). Antenatal couple counseling increases uptake of interventions to prevent HIV-1 transmission. J Acquir Immune Defic Syndr.

[20] Matovu JK, Todd J, Wanyenze RK, Wabwire-Mangen F, Serwadda D (2015). Correlates of previous couples’ HIV counseling and testing uptake among married individuals in three HIV prevalence strata in Rakai, Uganda. Glob Health Action.

[21] Wawer MJ, Gray RH, Sewankambo NK, Serwadda D, Paxton L, Berkley S, McNairn D, Wabwire-Mangen F, Li C, Nalugoda F (1998). A randomized, community trial of intensive sexually transmitted disease control for AIDS prevention, Rakai. Uganda. AIDS..

[22] Matovu JK, Gray RH, Makumbi F, Wawer MJ, Serwadda D, Kigozi G, Sewankambo NK, Nalugoda F (2005). Voluntary HIV counseling and testing acceptance, sexual risk behavior and HIV incidence in Rakai. Uganda. AIDS..

[23] Grabowski MK, Lessler J, Redd AD, Kagaayi J, Laeyendecker O, Ndyanabo A, Nelson MI, Cummings DA, Bwanika JB, Mueller AC (2014). The role of viral introductions in sustaining community-based HIV epidemics in rural Uganda: evidence from spatial clustering, phylogenetics, and egocentric transmission models. PLoS Med.

[24] Todd J, Carpenter L, Li X, Nakiyingi J, Gray R, Hayes R (2003). The effects of alternative study designs on the power of community randomized trials: evidence from three studies of human immunodeficiency virus prevention in East Africa. Int J Epidemiol.

[25] Gray RH, Kigozi G, Serwadda D, Makumbi F, Watya S, Nalugoda F, Kiwanuka N, Moulton LH, Chaudhary MA, Chen MZ (2007). Male circumcision for HIV prevention in men in Rakai, Uganda: a randomised trial. Lancet.

[26] Janz NK, Becker MH (1984). The health belief model: a decade later. Health Educ Q.

[27] Prochaska JO, DiClemente CC (1983). Stages and processes of self-change of smoking: Toward an integrative model of change. J Consult Clin Psychol.

[28] Dearing JW, Meyer G, Rogers EM, DiClemente RJ, Peterson JL (1994). Diffusion theory and HIV risk behavior change. Prevention AIDS: Theories and methods of behavioral intervention.

[29] Matovu JK, Wanyenze RK, Wabwire-Mangen F, Nakubulwa R, Sekamwa R, Masika A, Todd J, Serwadda D (2014). “Men are always scared to test with their partners … it is like taking them to the Police”: Motivations for and barriers to couples’ HIV counselling and testing in Rakai, Uganda: a qualitative study. J Int AIDS Soc.

[30] Lewin K (1947). Frontiers in group dynamics: concept, method and reality in social science; social equilibria and social change. Human Relations.

[31] Ministry of Health (2009). National couples’ HIV counseling and testing communication strategy 2009.

[32] Ministry of Health (2010). Uganda HIV counselling and testing policy.

[33] Conroy AA (2014). ‘It means there is doubt in the house’: perceptions and experiences of HIV testing in rural Malawi. Cult Health Sex.

[34] Campbell MK, Piaggio G, Elbourne DR, Altman DG (2012). Consort 2010 statement: extension to cluster randomised trials. BMJ..

[35] Kumwenda M, Munthali A, Phiri M, Mwale D, Gutteberg T, MacPherson E, Theobald S, Corbett L, Desmond N (2014). Factors shaping initial decision-making to self-test amongst cohabiting couples in urban Blantyre, Malawi. AIDS Behav.

[36] Thirumurthy H, Masters SH, Mavedzenge SN, Maman S, Omanga E, Agot K (2016). Promoting male partner HIV testing and safer sexual decision making through secondary distribution of self-tests by HIV-negative female sex workers and women receiving antenatal and post-partum care in Kenya: a cohort study. Lancet HIV.

[37] Martinez Perez G, Cox V, Ellman T, Moore A, Patten G, Shroufi A, Stinson K, Van Cutsem G, Ibeto M (2016). ‘I know that I do have HIV but nobody saw me’: oral HIV self-testing in an informal settlement in South Africa. PLoS One.

[38] Msuya SE, Mbizvo EM, Hussain A, Uriyo J, Sam NE, Stray-Pedersen B (2008). Low male partner participation in antenatal HIV counselling and testing in northern Tanzania: implications for preventive programs. AIDS Care.

[39] Lolekha R, Kullerk N, Wolfe MI, Klumthanom K, Singhagowin T, Pattanasin S, Sombat P, Naiwatanakul T, Leartvanagkul C, Voramongkol N (2014). Assessment of a couples HIV counseling and testing program for pregnant women and their partners in antenatal care (ANC) in 7 provinces, Thailand. BMC Int Health Hum Rights.

[40] Darbes LA, van Rooyen H, Hosegood V, Ngubane T, Johnson MO, Fritz K, McGrath N (2014). Uthando Lwethu (‘our love’): a protocol for a couples-based intervention to increase testing for HIV: a randomized controlled trial in rural KwaZulu-Natal, South Africa. Trials.

[41] Matovu JK, Kabanda J, Bwanika JB, Bwayo D, Asingwire N, Kyaddondo D, Coutinho SM (2014). Determinants of HIV counseling and testing uptake among individuals in long-term sexual relationships in Uganda. Curr HIV Res.

[42] Muhindo R, Nakalega A, Nankumbi J (2015). Predictors of couple HIV counseling and testing among adult residents of Bukomero sub-county, Kiboga district, rural Uganda. BMC Public Health.

[43] Tabana H, Doherty T, Rubenson B, Jackson D, Ekstrom AM, Thorson A (2013). ‘Testing together challenges the relationship’: consequences of HIV testing as a couple in a high HIV prevalence setting in rural South Africa. PLoS One.

[44] Vojnov L, Markby J, Boeke C, Harris L, Ford N, Peter T (2016). POC CD4 testing improves linkage to HIV care and timeliness of ART initiation in a public health approach: a systematic review and meta-analysis. PLoS One.

[45] Njau B, Damian DJ, Abdullahi L, Boulle A, Mathews C (2016). The effects of HIV self-testing on the uptake of HIV testing and linkage to antiretroviral treatment among adults in Africa: a systematic review protocol. Systematic Reviews.

[46] Ministry of Health (2005). Uganda national HIV counseling and testing policy.

[47] Wagman JA, Gray RH, Campbell JC, Thoma M, Ndyanabo A, Ssekasanvu J, Nalugoda F, Kagaayi J, Nakigozi G, Serwadda D (2015). Effectiveness of an integrated intimate partner violence and HIV prevention intervention in Rakai, Uganda: analysis of an intervention in an existing cluster randomised cohort. Lancet Glob Health.

[48] Grosskurth H, Mosha F, Todd J, Mwijarubi E, Klokke A, Senkoro K, Mayaud P, Changalucha J, Nicoll A, Ka-Gina G (1995). Impact of improved treatment of sexually transmitted diseases on HIV infection in rural Tanzania: randomised controlled trial. Lancet.

[49] Dandona L, Kumar GA, Lakshmi V, Ahmed GM, Akbar M, Ramgopal SP, Sudha T, Alary M, Dandona R (2013). HIV incidence from the first population-based cohort study in India. BMC Infect Dis.

[50] Casanova L, Gobin N, Villani P, Verger P. Bias in the measure of the effectiveness of seasonal influenza vaccination among diabetics. Prim Care Diabetes. 2016. http://dx.doi.org/10.1016/j.pcd.2016.05.005. 10.1016/j.pcd.2016.05.005 27290610

[51] Reusch A, Strobl V, Ellgring H, Faller H (2011). Effectiveness of small-group interactive education vs. lecture-based information-only programs on motivation to change and lifestyle behaviours. A prospective controlled trial of rehabilitation inpatients. Patient Educ Couns.

[52] Garrett N, Hageman CM, Sibley SD, Davern M, Berger M, Brunzell C, Malecha K, Richards SW (2005). The effectiveness of an interactive small group diabetes intervention in improving knowledge, feeling of control, and behavior. Health Promot Pract.

